# Randomized controlled trial of a computer-tailored multiple health behaviour intervention in general practice: 12-month follow-up results

**DOI:** 10.1186/1479-5868-11-41

**Published:** 2014-03-19

**Authors:** Sanjoti Parekh, David King, Frances M Boyle, Corneel Vandelanotte

**Affiliations:** 1 School of Population Health, The University of Queensland, Herston, Queensland 4006, Australia; 2 School of Medicine, The University of Queensland, Herston, Queensland, Australia; 3 Head of Social Science & Health Systems Group, School of Population Health, The University of Queensland, Herston, Queensland, Australia; 4 Director, Centre for Physical Activity Studies, NHF & NHMRC Research Fellow, Institute for Health and Social Science Research Central Queensland University, Rockhampton, Queensland, Australia

**Keywords:** General practice, Prevention, Non-communicable disease, Computer-tailored, Randomised controlled trial

## Abstract

**Background:**

Effective strategies to address risk factors of non-communicable diseases are required to curtail the expanding costs of health care. This trial tested the effectiveness over one year of a minimal intervention targeting multiple health behaviours (diet, physical activity, alcohol and smoking) in a general practice setting, through the provision of personalised, computer-tailored feedback.

**Methods:**

Patients who had attended a general practice in the previous 6 months were recruited from 21 general practitioners in Brisbane, Australia. Baseline data were collected using self-reports on adherence to ten health behaviours and summarised into a health score from 0 to 10. This randomised controlled trial used a 2×2 factorial design, with one arm randomising subjects to the intervention or control group. The other arm was either feedback at baseline (single contact) or an additional assessment with feedback at 3 months (dual contact). As such, 4 study groups created were, to which participants were randomised blindly: A. Intervention with single contact; B. Intervention with dual contact; C. Control with single contact and D. Control with dual contact. All participants were assessed again at 12 months.

**Results:**

Of the 4676 participants randomised, 3065 completed questionnaires at 12 months. Both single and dual contact groups improved their 10 item health scores (+0.31 and +0.49 respectively) relative to control group outcomes (+0.02; p < 0.01). Improvement in adherence to guidelines for fish intake, type of milk consumed, vegetable and fruit intake, and alcohol intake were observed in single and dual contact intervention groups (p < 0.01). Both intervention groups showed greater improvement than controls for individual health behaviours, apart from red meat intake, smoking behaviour, physical activity and body weight. Interestingly, there was an improvement in reported non-smoking rates in both intervention and control groups (3% single contact; 4.5% dual contact).

**Conclusions:**

Small but meaningful long-term changes in health behaviours can be achieved with a low-intensity intervention, which may reduce health care costs if implemented on a large scale. Further research is needed to better understand the mechanism by which maintenance of behaviour change can be achieved.

**Trial Registration:**

The Australian New Zealand Clinical Trials Registry: ACTRN12611001213932.

## Background

A set of common risk factors including unhealthy diet, insufficient physical activity, excessive alcohol intake and smoking is linked with multiple non-communicable diseases (NCDs) [1]. Ideally these multiple behavioural health risk factors could be addressed simultaneously but little research has examined the efficacy of such interventions [2]. As a result, significant challenges remain in selecting the optimal mix of strategies for prevention of NCDs, particularly at the population-wide level.

General practitioners (GPs) are ideally placed to contribute to the prevention of NCDs, as the majority of Australians visit a GP each year [3]. However, many barriers to GP involvement in addressing health behaviours have been identified, with lack of time featuring prominently [4]. Prevention strategies that are minimally disruptive to routine patient care and place no additional burden on GPs are needed. Computer-tailored health promotion interventions that provide patients with personal feedback meet these criteria and have shown promising results [5]. Tailored communications have been reported as better remembered, more often read, and perceived as more relevant or credible compared with non-tailored communication [6]. Tailored approaches developed using computer-based algorithms can be used repeatedly to reach large groups with little effort and are potentially cost-effective [7].

Evidence that multiple health behaviours can be summed into a single, composite score to estimate overall impact on health [8-10] might assist interventions to focus on multiple behaviours simultaneously. In a cohort of 12,203 elderly men, Spencer *et al*. showed that a simple score summarising eight health behaviours had a significant predictive ability for mortality from all causes over five years [11,12]. Research that focuses on both clustering of health behaviours and adherence is scarce though clustering of healthy behaviours may be associated with greater uptake of clinical preventive practices [13]. For many individual health behaviours, including dietary patterns [14,15], physical activity [16], salt intake [17] and weight loss [18-20] long term adherence is found to be poor without support and reinforcement.

Combining various strategies such as addressing multiple lifestyle factors concurrently, delivery through general practice, applying a combined score to summarise health behaviours and providing computer-tailored advice offers a pragmatic approach to promoting long-term lifestyle behaviour change that has the ability to be applied on a population-wide scale. In our previous research we developed a validated data collection tool that assesses and summarises multiple health behaviours using a single “Prudence Score” [21]. A computer-tailored intervention (10 Small Steps) was developed to provide feedback derived from this summary score [22] and its effectiveness at 3 months has been reported [23]. The aim of the current study is to report on the longer-term outcomes of the intervention; specifically, (a) to assess the effectiveness of the 10 Small Steps intervention after 12 months, and (b) to determine whether additional reinforcement at 3 months improves outcomes at 12 months.

## Methods

### Overview of design

**Figure 1:**
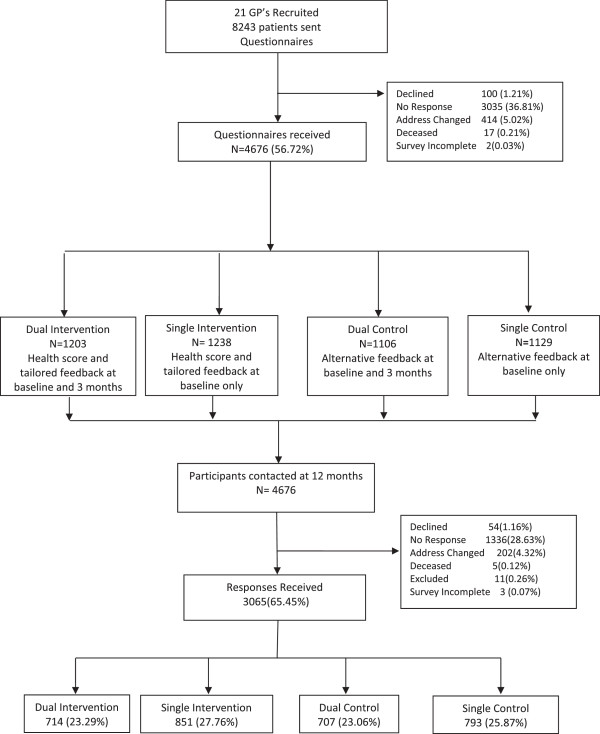
Flowchart of participant recruitment and randomisation.

In 2008, 30 GPs in metropolitan Brisbane, Australia, were invited to participate in the 10 Small Steps study. The study protocol has been detailed elsewhere (21). Briefly, participating GPs nominated all eligible patients aged between 18 to 70 years who had consulted them in the previous six months. Patients with active cancer, receiving renal dialysis, recent cardiovascular event, dementia, any other terminal illness or recent bereavement were excluded. Names and addresses of eligible patients were provided to the research team, who sent each patient a written invitation to participate, together with a baseline questionnaire and reply-paid envelope. The GP’s letterhead and electronic signature was used for this correspondence. Non-responders were sent up to two reminder letters and a new copy of the questionnaire at two weekly intervals. Completion and return of the questionnaire was regarded as consent to participate. Participants could decline involvement in the study at any stage. Figure 1 outlines the participant flow.

For the trial, survey respondents were randomised using a permuted block procedure stratified by GP. Participants residing at the same address were allocated to the same group to avoid contamination across groups. This randomised controlled trial used a 2×2 factorial design, with one arm randomising subjects to the intervention of feedback on combined health score and personalised computer tailored advice, or a control group receiving alternative feedback. The other arm was either feedback at baseline (single contact) or an additional assessment and computerised feedback at 3 months (dual contact). The 4 study groups created were (A) Intervention with single contact; (B) Intervention with dual contact; (C) Control with single contact and (D) Control with dual contact. The participants were blinded to the group to which they were randomised. All participants were assessed again at 12 months with self-reporting of the 10 health behaviours.

Ethics approval was obtained from the Behavioural and Social Sciences Ethical Review Committee of The University of Queensland, Australia. This trial is registered under The Australian New Zealand Clinical Trials Registry: ACTRN12611001213932.

### Measurement Instrument

The baseline questionnaire has been validated [21] and includes 26 questions related to ten health behaviours and nine questions collecting demographic information. Responses to items addressing smoking, physical activity (short International Physical Activity Questionnaire [24]), intake of alcohol, meat, fish, vegetables and fruit (V&F), use of unsaturated fats as spreads, avoidance of added salt, type of milk consumed, and body mass index (BMI) were dichotomised. Each behavioural item was assigned a score of '1’ if adhering to health recommendations or '0’ when not adhering to health recommendations. Scores were based on guidelines promulgated by the National Health and Medical Research Council (NHMRC) and the National Heart Foundation of Australia (NHF). Individual health behaviour scores were summed to yield a combined score ranging from 0 to 10. The remaining items in the baseline questionnaire addressed other health behaviours such as tetanus immunization, sun protection behaviour, non-smoking policies in the home, and participation in mammography and cervical cytology screening. These items did not contribute to the combined score, but were used to provide the control groups with alternative feedback and minimise the risk of attrition.

### Intervention content

The information material for intervention group participants consisted of:

(1) *Personalised computer*-*tailored feedback*: This focused on six dietary behaviours as well as responses to smoking behaviour, alcohol intake, physical activity and BMI. A one-page, personalised, computer-tailored feedback letter indicating the participant’s combined health score and behaviours not meeting guideline recommendations. This letter was printed on the treating GP’s letterhead and encouraged the adoption of at least one behaviour not already contributing to the participant’s Prudence Score. The decision as to which additional behaviour(s) to improve was the patient’s own.

(2) *Health Promotion Information Material*: One-page health promotion information sheets were distributed to participants only for behaviours not meeting national guidelines. For example, participants who did not meet NHMRC guidelines for vegetable intake but did meet recommendations for fruit intake only received the information sheet related to daily vegetable intake.

The information material for control group participants consisted of:

(1) *Personalised computer*-*tailored feedback*: This focused on 5 other health protective behavaiours (tetanus immunization, sun protection behaviour, non-smoking policies in the home, and participation in mammography and cervical cytology screening). A one-page, personalised, computer-tailored feedback letter indicating behaviours that meet guideline recommendations was provided. This letter was printed on the treating GP’s letterhead and encouraged the adoption of at least one behaviour not already followed by the participant.

(2) *Health Promotion Information Material*: One-page health promotion information sheets were distributed to participants only for behaviours not meeting national guidelines for 5 listed behaviours. For example, participants who did not meet Cancer Council guidelines for sun protection behaviour but did meet recommendations for non-smoking policies at home only received the information sheet related to sun protection.

### Follow-up

The same assessment questionnaire used at baseline was posted to participants at 3 and/or 12 months. Non-responders were sent up to two reminders along with copies of the cover letter and questionnaire at two week intervals following the initial mailing.

### Analysis

All analyses were pre-planned and based on the primary aims of the study. The completed surveys that had more than 10% of missing data were excluded from the study. Baseline differences in groups were analysed with one-way ANOVA for continuous variables and Chi squared test for categorical variables. Participants were classified by their study group and initial analysis performed to examine comparability across baseline Prudence Score, age, gender, marital status, employment status, education level and area of residence. Participants were compared with non-responders at each follow-up period.

The primary analysis compared change in Prudence Scores of the intervention group over and above the change in control group at 12 months using General Estimating Equations Models. Change in this score was also calculated for dual and single contact groups again using General Estimating Equations Models. Change in individual health behaviours at 12 months was also examined across the four study groups. The per-protocol analysis was performed along with the intention-to-treat analysis which was based on the assumption that non-completers did not change their behaviour in any direction. Hence all the data collected at baseline, 3 months and 12 months was analysed using General Estimating Equations Models adjusted for age and educational status. Significance was set at *P* < 0.05 for all analyses.

Power calculations for the main study were based on pilot study results [21] (mean Prudence Score 4.94, SD 1.7). To have a 95% chance of the proportion with a Prudence score of 6 or more increase from 39% to 45%, using two-sided α = 0.05, required a total of 6600 invitations to participate, accounting for 20% loss to follow up and response fraction of 60% achieved in the pilot study.

## Results

Twenty-one of the thirty invited GPs agreed to participate. In total 8243 patients of these GPs were included in the list of eligible patients. 4678 participants returned questionnaires at baseline and 3068 completed the self-reported questionnaire at 12 months. However, two surveys at baseline and three surveys at 12 months had more than 10% missing data, hence were excluded. Therefore, the number of participants at baseline was 4676 and at 12 was 3065 (completion fraction: 65.45%) (Figure 1).

### Demographics

Complete data were available for 2873 participants. There were no baseline differences in Prudence Score or socio-demographic variables between intervention and control group participants who responded at 12 months (Table 1). Mean age of participants was 46.9 years (CI 46.5-47.3) and 69% were women. A high percentage of participants were tertiary-educated (58.7%), married (71.7%) and employed (65.2%). The distribution of Prudence Score was Gaussian and the modal Prudence Score was 5 to 6 (44.5%) on the scale of 0 to 10. Non-respondents at both 3 and 12 months were significantly older and reported lower educational attainment (Table 2). Hence all analyses assessing adherence to health behaviours were adjusted for age and educational status.

**Table 1:** Baseline characteristics for 12 months follow-up respondents

**Behaviour and other socio demographics**	**All**	**Intervention**	**Control**
	**N (%)**	**N (%)**	**N (%)**
**Prudence Score**			
0-2	29 (1.1)	17 (1.99)	12 (0.9)
3-4	448 (16.7)	230 (16.8)	218 (16.7)
5-6	1189 (44.5)	612 (44.7)	577 (44.2)
7-8	886 (33.2)	449 (32.8)	437 (33.5)
9-10	121 (4.5)	60 (4.4)	61 (4.7)
**Age group**			
18 to 39	674 (23.5)	343 (23.4)	331 (23.6)
40 to 59	1364 (47.6)	688 (46.9)	676 (48.3)
60+	829 (28.9)	436 (29.7)	393 (28.1)
**Gender**			
Male	884 (30.8)	468 (31.9)	416 (29.6)
Female	1988 (69.2)	1000 (68.1)	988 (70.4)
**Education**			
Uni and diploma	1682 (58.7)	885 (60.4)	797 (57.1)
High school and below	1181 (41.3)	581 (39.6)	600 (42.9)
**Marital status**			
Married	2056 (71.7)	1067 (72.8)	989 (70.6)
Single	809 (28.3)	398 (27.2)	411 (29.4)
**Employment status**			
Employed	1869 (65.2)	943 (64.3)	926 (66.2)
Not employed	996 (34.8)	523 (35.7)	473 (33.8)
**Area of residence**			
Affluent	1262 (44.0)	654 (44.6)	608 (43.4)
Disadvantaged	905 (31.6)	452 (30.8)	453 (32.4)
Most disadvantaged	700 (24.4)	361 (24.6)	339 (24.2)

Total number of participants vary for each characteristic due to missing data.

**Table 2:** Socio-demographic differences for respondents and non-respondents at 3 and 12 months follow-up

	**3 months participants (n = 2309)**			**12 months participants (n = 4676)**		
	**Respondents N = 1711**	**Non-respondents N = 598**	**p-value^**	**Respondents N = 3065**	**Non-respondents N = 1611**	**p-value^**
Gender (% men)	30.3	34.9	0.07	30.9	33.1	0.15
Age (% > 50 years)	50.2	68.4	0.00	49.1	67.4	0.00
Education (% university or diploma holders)	57.5	51.8	0.02	58.1	54.7	0.05
SES (% affluent)	43.8	48.6	0.06	44.1	47.4	0.12

^Chi2 test used to calculate p-value for significant difference in respondents and non-respondents for specific variable.

### Change in prudence score

Table 3 compares change in Prudence Score for the dual and single contact groups. From 1421 participants in dual contact and 1644 in single contact group; complete data were available for 1328 dual contact and 1545 single contact participants. In the per protocol analysis a greater change was observed at 12 months for participants who received dual contact. (difference of +0.49) compared to participants receiving single contact (difference of +0.31); however this difference was not significant (coefficient 0.02 [CI = -0.07 to 0.11] & p = 0.70). The changes in mean Prudence Score over 12 months between intervention and control groups were significant even after controlling for baseline differences: for single contact (coefficient 0.16 [CI = 0.09 to 0.23]; p < 0.01) and dual contact (coefficient 0.17 [CI = 0.10 to 0.24]; p < 0.01) groups. For men the change in Prudence Score from baseline was smaller than for women, and of similar size in both dual and single contact groups (Table 3). For women, the increase in Prudence Score at 12 months for intervention group was larger in dual versus single contact group (+0.54 vs. +0.32; p = 0.33).

**Table 3:** Change in mean Prudence Score at 12 months

	**Dual contact group**			**Single contact group**			**Coefficient^ and p-value**
	**Baseline**	**12 months**	**Net change**	**Baseline**	**12 months**	**Net change**	
**All (n = 2872)**							
Intervention	5.78 (5.69-5.88)	6.27 (6.15-6.39)	+0.49	5.82 (5.72-5.91)	6.13 (6.03-6.24)	+0.31	0.02 (-0.07-0.11) p = 0.70
Control	5.87 (5.77-5.97)	5.89 (5.77-6.01)	+0.02	5.77 (5.67-5.87)	5.93 (5.82-6.04)	+0.16	
**Coefficient^**	0.17 (0.10-0.24) , p < 0.01			0.16 (0.09-0.23), p < 0.01			
**Men (n = 884)**							
Intervention	5.62 (5.46-5.78)	6.00 (5.77-6.22)	+0.38	5.72 (5.57-5.88)	6.04 (5.86-6.22)	+0.32	0.04 (-0.21-0.13) p = 0.64
Control	5.70 (5.52-5.87)	5.66 (5.47-5.87)	-0.04	5.54 (5.36-5.71)	5.57 (5.38-5.77)	+0.03	
**Coefficient^**	0.25 (0.8-0.42), p < 0.01			0.16 (0.5-0.18), p < 0.01			
**Women (n = 1988)**							
Intervention	5.87 (5.75-5.98)	6.39 (6.25-6.53)	+0.52	5.86 (5.74-5.97)	6.18 (6.05-6.37)	+0.32	0.06 (-0.06-0.18) p = 0.33
Control	5.94 (5.83-6.06)	5.98 (5.84-6.13)	+0.04	5.87 (5.75-5.98)	6.08 (5.95-6.21)	+0.21	
**Coefficient^**	0.22 (0.13-0.31), p < 0.01			0.15 (0.07-0.24), p < 0.01			

^Generalised estimating equations used to calculate coefficient and p-value.

Analysis adjusted for age and education status.

An intention-to-treat analysis was undertaken assuming that the participants who did not reply at 12 months did not change their baseline Prudence Score. The analysis showed smaller but still significant change in the mean Prudence Score for the intervention group compared to the control group (t = 3.43, p = 0.001). For the dual contact group difference between intervention and control mean Prudence Score was +0.15 and for the single contact group it was +0.22, showing again no statistically significant difference between dual and single contact groups at 12 months (p = 0.22).

### Change in individual health behaviours

The percentage change in adherence to ten individual health behaviours in the dual and single contact groups are shown in Table 4 and Table 5. At baseline there were no significant differences between groups for any of the ten behaviours. At 12 months both dual and single intervention participants had significantly improved (when compared to their respective control groups) five out of ten behaviours. None of the groups showed statistically significant improvement in meat intake, smoking, physical activity or BMI at 12 months after the intervention. However, both dual intervention and dual control groups showed positive improvement (+4.48% dual intervention; +4.42% dual control) in adherence to smoking guidelines. Statistically significant odds ratios reflecting improvements in fish, milk, V&F, and alcohol were common to both intervention groups, with the single group showing an improvement in type of spread used, and the dual group showing improved adherence to recommended guidelines for salt intake. The increase in adherence to guidelines for some behaviours was considerable, especially in the dual intervention group: for example, fish intake (+11.87%), V&F intake (+9.23%) and safe alcohol consumption levels (+10.42%).

**Table 4:** Percentage of dual contact participants adhering to individual health behaviours (n = 1328)

	**Baseline**			**12 months**			**% difference within groups (12 months minus baseline %)**		**Difference between intervention & control group change at 12 months#**	
	***Intervention %***	***Control %***	***p-value^***	***Intervention %***	***Control %***	***p-value^***	***Intervention***	***Control***	***OR (95% CI)***	***p-value***
Meat	72.2	74.5	0.21	67.0	70.1	0.21	-5.2	-4.4	0.90 (0.82-1.0)	0.08
Fish	67.0	69.3	0.25	78.9	73.2	0.01*	+11.9	+3.9	**1.38 (1.22**-**1.55)**	0.00*
Milk	68.9	70.2	0.52	75.1	73.1	0.39	+6.1	+2.9	**1.15 (1.06**-**1.26)**	0.01*
Salt	44.8	44.4	0.83	52.4	45.3	0.01*	+7.6	+0.9	**1.14 (1.06**-**1.24)**	0.01*
V&F	11.9	12.1	0.89	21.2	12.7	0.00*	+9.2	+0.6	**1.37 (1.18**-**1.59)**	0.00*
Spread	66.4	66.3	0.95	72.6	64.7	0.00*	+6.1	-1.7	1.08 (0.99-1.18)	0.10
Smoking	84.5	86.0	0.31	89.0	90.4	0.38	+4.5	+4.4	1.04 (0.96-1.14)	0.14
Physical activity	49.9	53.0	0.13	48.7	46.8	0.48	-1.2	-6.2	0.85 (0.76-1.01)	0.10
Alcohol	67.9	69.7	0.35	78.3	75.5	0.20	+10.4	+5.8	**1.34 (1.21**-**1.48)**	0.00*
Body weight	43.5	38.8	0.06	42.3	39.7	0.34	-1.2	+1.7	1.02 (0.95-1.10)	0.49

^Chi2 used to test the significant difference for % adherence between intervention and control groups.

#GEE used for measuring statistical significance for difference between changes in intervention group over and above the change in control group.

*Significant results (Bolded Text).

Analysis adjusted for age and education status.

V&F: Vegetable and Fruit.

**Table 5:** Percentage of single contact participants adhering to individual health behaviours (n = 1545)

	**Baseline**			**12 months**			**% difference within groups (12 months minus baseline %)**		**Difference between change in intervention & control at 12 months#**	
	***Intervention %***	***Control %***	***p^***	***Intervention %***	***Control %***	***P^***	***Intervention***	***Control***	***OR***	***p-value***
Meat	70.9	71.8	0.62	70.3	72.1	0.27	-0.6	+0.3	0.97 (0.88-1.08)	0.68
Fish	68.5	67.8	0.27	74.4	71.7	0.225	+5.9	+3.9	**1.26 (1.10**-**1.45)**	0.00*
Milk	68.5	69.5	0.59	72.7	71.8	0.553	+4.2	+2.3	**1.15 (1.05**-**1.25)**	0.00*
Salt	41.6	42.8	0.54	46.3	42.7	0.136	+4.8	-0.2	1.05 (0.97-1.14)	0.19
V&F	13.9	11.2	0.10	18.8	11.9	0.000*	+4.9	+0.7	**1.22 (10.6**-**1.41)**	0.00*
Spread	67.5	65.1	0.223	71.2	67.9	0.159	+3.6	+2.8	**1.12 (1.02**-**1.23)**	0.01*
Smoking	87.3	86.7	0.65	90.2	89.8	0.63	+2.9	+3.1	1.06 (0.95-1.18)	0.23
Physical activity	52.4	50.4	0.348	51.8	47.3	0.06	-0.5	-3.1	0.91 (0.82-1.02)	0.12
Alcohol	68.4	69.5	0.605	76.9	74.4	0.246	+8.5	+4.9	**1.37 (1.19**-**1.57)**	0.00*
Body weight	40.7	40.7	0.98	41.7	41.9	0.923	+1.0	+1.2	1.06 (0.92-1.21)	0.37

^Chi2 used to test the significant difference for % adherence between intervention and control groups.

#GEE used for measuring statistical significance for difference between changes in intervention group over and above the change in control group.

*Significant results (Bolded Text).

Analysis adjusted for gender and education status.

V&F: Vegetable and Fruit.

## Discussion

The 10 Small Steps project demonstrates that a computer tailored, multiple health behaviour intervention can be implemented successfully [22,23] and improve adherence to healthy behaviours over a 12-month period. The intervention group sustained behaviour changes following a non-contact period of 9 or 12 months. While the dual contact intervention did not result in a statistically significant benefit over the single contact intervention for the former group there was a trend to larger change in some individual behaviours. Importantly, a significant positive change remained in both groups compared to their respective control groups at 12 months.

This intervention trial combined a number of strategies that individually have shown to be important in improving health behaviours in the context of primary care. These include: the use of low-intensity computer-tailored feedback [5], simultaneous focus on multiple health behaviours [25], endorsement of GPs [26,27], and the use of an intervention reinforcement [28]. A direct comparison of the outcomes of the current study with other studies is not possible, as other studies have incorporated some but not all of these elements. For example, a low intensity intervention in a primary care setting for reducing fat and fibre intake was effective after 12 months [29,30]. Endorsement of the intervention by practitioners was proposed as the possible reason for long term success [29]. A number of primary care interventions applying computer-tailored approaches have demonstrated effectiveness, though they were focused on single risk factors, intensive (including more than one or two sessions with computer-tailored advice) or focused on secondary prevention only [31-33]. Another successful computer-tailored intervention addressed two behaviours simultaneously but was more comprehensive in content [34].

Many computer-tailored interventions targeting behaviours, show intervention effects declining quickly after intervention completion, despite initial effectiveness [35,36]. Notably, this was not the case for the '10 Small Steps’ study. At 12 months the participants still showed significant improvements in the health behaviours suggesting that the inclusion of additional strategies alongside computer tailoring, such as the endorsement of GPs, may have added benefits in bringing about and maintaining behaviour change. It is difficult to attribute the positive outcomes observed in the '10 Small Steps’ study to any individual strategy and the synergistic effects of the above proven strategies are likely to be responsible for underlying success in promoting adoption and maintenance of health behaviours in this study.

Most studies evaluating long-term effectiveness of interventions indicate that maintenance of healthy behaviours is difficult. Smoking cessation, for example, has been shown to require a comprehensive and intensive personally tailored approach to help smokers quit in the long term [37]. Similarly, a systematic review of physical activity interventions suggested that additional tailored exercise prescription strategies and booster interventions such as via phone, mail or internet were needed to facilitate long-term (at least 12 months) effectiveness [38]. This lack of intensity in our pragmatic intervention may explain the failure to achieve significant improvements in 'difficult to change’ behaviours such as physical activity, smoking and BMI. However, all intervention and control groups returned positive changes of 3 to 4% in smoking behaviour, which is significant. This indicates that even the 'control group’ received an intervention, simply by completing a health assessment questionnaire sent with approval of their GP, even without additional focused and tailored feedback. Background smoking rates are declining in Australia, but not to this extent in one year. From 1991 to 2004 the prevalence of smoking in Australia fell from 27.1% to 19.0%, a fall of 8.1% in absolute terms, averaging 0.58% per year decline in smoking, which is far less than the observed decline in this study [39,40].

There were noticeable similarities in dual and single contact groups: increased fish intake, increased use of low fat milk, increased V&F intake and a higher proportion of participants drinking alcohol within guidelines. However, there were some discrepancies between the intervention groups as well: while the single contact group increased the use of spreads other than butter, this was not the case with the dual contact group. The dual contact reduced use of salt which was not the case with the single contact group. It is possible that certain behaviours such as salt intake are habitual and require repeated efforts to change. Habit formation theory posits that habit strength increases as a result of repetition and positive reinforcement and that any type of repetitive behaviour requires decreasing mental effort before eventually becoming habitual [41,42]. In our study, participants in the dual contact group who reduced salt intake might also have been successful in changing other behaviours during 12 months of intervention. The inconsistencies in the adoption of various behaviours in our study suggest that the role of habit formation in relation to behaviour change needs to be further explored. It is also possible that there is a limit to how much an individual can change and, as a result, individuals may “swap” habits by, for example, giving up one healthy habit to take up another that is perceived as more important. There are studies illustrating the limitations of self-regulatory capacity and the operating of concepts such as decision fatigue, indicating that it might be difficult for an individual to make multiple behavioural changes simultaneously [43]. However, attention to simultaneous or multiple-behaviour change is likely to have a greater impact on public health than sequential or single-behaviour change, possibly by giving individual the autonomy to choose behaviours they perceive as easiest to change. This process can build self-efficacy to change other unhealthy behaviours. Research also suggests simultaneous interventions are more cost effective than sequential interventions which require more resources to repeatedly reach out to participants [34].

Limitations of our study include the use of a dichotomous scoring system for health behaviours, where relevant sub-threshold change in behaviour remains undetected. Thus our results may have underestimated the real extent of behaviour change. Secondly, the ten component behaviours of the Prudence Score are equally weighted, rather being weighted according to their relative impacts on health. Also, dietary factors are over represented compared to exercise and smoking, which only contributed a single score each. Assessing the impact on morbidity and mortality is beyond the scope of this trial. However, a study employing an equally weighted lifestyle scoring system using all the same items as the Prudence Score (except vegetable and fruit intake and type of spread ) was able to predict mortality in both healthy elderly men and elderly men with established vascular disease [12,44]. This suggests that the aggregate unweighted score is still a meaningful summary of an individual’s effort to protect their health. As change in health behaviours was examined in two ways (as individual behaviours and as a sum score) it is possible that some significant findings might be attributed to conducting multiple analyses. Thirdly, the use of self-reported data was a potential weakness despite using a previously validated assessment questionnaire [21]. Whilst some behaviour can be monitored objectively, for example physical activity levels, this is problematic for most dietary behaviours, and alternatives are either extremely costly or impractical. However, the same survey instrument was used to assess behaviours before and after interventions. Another limitation of this study is its inability to undertake a cost-effectiveness analysis as it was not one of the planned outcomes of the trial. However, it is important that future studies consider such analysis.

Although the results of this study do not show a major shift in health behaviours, small individual level changes can be meaningful and contribute to reducing the burden of chronic disease on a population level if the intervention is implemented on a large scale. Composite health scores that showed significant improvements are associated with better health [9,45] and successful aging [46]. The concept of changing health score on the population wide scale is in keeping with Geoffrey Rose’s public health approach, focusing on shifting the distribution of population risk exposure toward a lower mean rather than simply focussing on high risk individuals [47,48].While the issue of optimal balance between targeted high risk strategies and wider population health strategies is often debated [49], it is important that these strategies work synergistically. Targeting patients using bio-markers can help identifying high risk population whereas using a simple but comprehensive lifestyle behaviour score such as the Prudence Score can be helpful in addressing determinants of ill health for the entire populations. Such lifestyle scores do not rely on biological tests and are easy to comprehend by the lay public. Moreover, interventions such as 10 Small Steps have the ability to work as a trigger for practitioners to briefly and confidently discuss health risk behaviours with their patients, further enhancing behavioural outcomes.

A key strength of this study was the combination of a number of behaviour change strategies known to be both effective and relevant in the primary care setting. It targeted multiple behaviours in a large sample and assessed for long term change with and without reinforcement. Whilst developing a computer-tailored intervention is initially costly and time consuming, such expert-systems can be cost-effective in the longer term and implementable on a large scale [35]. The simplicity of the intervention itself implies a greater feasibility for translation into practice, a step previously effective interventions have failed due to complexity or effort required to implement. Finally, participants were drawn from general practice and representative of the wider Australian population [3,50], so the results of this study can be generalised to the primary care setting.

## Conclusions

The challenge for reducing NCDs lies in translating current knowledge about risk factors into meaningful and sustainable behaviour change. This requires feasible strategies that go beyond simply communicating messages to facilitating and empowering individuals to adopt and maintain healthy behaviours. This trial provides evidence that long-term changes in health behaviours can be achieved with a low-intensity intervention. Previous research has established that combined health scores are well correlated with morbidity and mortality. This research used the combined health score to positively motivate behaviour change, in concert with other behaviour change strategies in its design. Further research is needed to better understand the mechanism by which behaviour change and maintenance is achieved and which attributes of this intervention promoted improved health behaviours.

## Competing interests

The authors declare that they have no competing interests.

## Authors’ contributions

SP recruited the general practitioners and study participants, collected and analysed the data and drafted the manuscript. DK participated in recruiting general practitioners. CV also provided supervision in generating tailored feedback and health promotion materials. DK, FB and CV helped to draft the manuscript. KJ conceived the study, and contributed to its design prior to his death in 03/2010. SP, CV, DK, and FB read and approved the final manuscript.
